# Risk factors associated with birth asphyxia at Pumwani Maternity Hospital, Nairobi, Kenya: a case-control study

**DOI:** 10.3389/fped.2026.1886479

**Published:** 2026-09-04

**Authors:** Ruth Wagathu, James Orwa, Peterson Kiraithe, Abednego Ongeso, Evah Maina, David Gerald Omondi, Benard Mutwiri, Timothy Guetterman

**Affiliations:** 1 School of Nursing and Midwifery, Aga Khan University, Nairobi, Kenya; 2 Department of Population Health, Aga Khan University, Nairobi, Kenya; 3 School of Pharmacy and Health Sciences, United States International University, Nairobi, Kenya; 4 Department of Family Medicine, University of Michigan, Ann Arbor, MI, United States

**Keywords:** birth asphyxia, case-control study, fetal hypoxia, Kenya, newborn, risk factors

## Abstract

**Background:**

Birth asphyxia remains a leading cause of neonatal mortality and morbidity in low- and middle-income countries. In Kenya, it accounts for approximately 29% of neonatal deaths despite the implementation of national newborn care guidelines. This study investigated the risk factors associated with birth asphyxia at Pumwani Maternity Hospital, Nairobi, Kenya.

**Methods:**

A matched case-control study was conducted at Pumwani Maternity Hospital, Nairobi, Kenya, between February and September 2024. Cases were newborns with a five-minute APGAR score <7, while controls had a five-minute APGAR score ≥7. A total of 540 newborns (270 cases and 270 controls) were consecutively recruited. Data were collected using a researcher-administered questionnaire and a data abstraction tool. Univariable and multivariable conditional logistic regression analyses were performed to identify factors associated with birth asphyxia. All analyses were conducted using R version 4.5.1 (2025-06-13 ucrt), and statistical significance was set at *p* < 0.05.

**Results:**

Most mothers were aged above 20 years (80.2%), married or cohabiting (75.6%), unemployed (61.9%), and had attained secondary level education (58.0%). Complications during labour were strongly associated with birth asphyxia (AOR: 24.26; 95% CI: 7.65–76.95). Additionally, previous adverse obstetric history (AOR: 2.18; 95% CI: 1.14–4.17), maternal medical and obstetric conditions during pregnancy (AOR: 1.82; 95% CI: 1.04–3.19), and non-cephalic fetal presentation (AOR: 8.92; 95% CI: 1.77–45.00) were also independently associated with higher odds of birth asphyxia. Conversely, spontaneous onset of labour (AOR: 0.20; 95% CI: 0.05–0.76), spontaneous vaginal delivery (AOR: 0.02; 95% CI: 0.00–0.10), and delivery conducted by trained healthcare providers were associated with reduced odds of birth asphyxia.

**Conclusion:**

Birth asphyxia was strongly associated with several maternal and obstetric factors. Strengthening antenatal risk identification, timely management of labour complications, skilled intrapartum care, and early recognition of high-risk deliveries may reduce the burden of birth asphyxia and improve neonatal outcomes in similar low-resource settings.

## 1 Introduction

Birth asphyxia is defined by the World Health Organization (WHO) as the inability of a newborn to initiate and sustain spontaneous breathing at birth (1). The condition results from impaired placental or pulmonary gas exchange during the perinatal period, leading to hypoxia, hypercapnia, and metabolic acidosis, which, if prolonged, may progress to hypoxemia and multisystem organ dysfunction (2–4). In clinical practice, a five-minute APGAR score of less than seven is commonly used as an operational indicator of birth asphyxia, particularly in epidemiological studies and resource-limited settings (5, 6). However, the APGAR score alone is insufficient to establish the diagnosis because it may be influenced by factors other than intrapartum hypoxia (7). A comprehensive diagnosis therefore incorporates additional clinical and laboratory findings including umbilical cord arterial pH below 7.0, evidence of neonatal encephalopathy like seizures, prolonged need for resuscitation, and multisystem organ dysfunction (3, 8).

Globally, birth asphyxia is responsible for approximately 23% of neonatal deaths, making it the second leading cause of neonatal mortality and among the top five causes of death in children under five years of age (2, 9, 10). While developed countries have substantially reduced the burden of birth asphyxia through advances in obstetric and neonatal care, low- and middle-income countries continue to experience disproportionately higher rates (3, 4, 11). Across East Africa, birth asphyxia remains a major contributor to neonatal morbidity and mortality despite ongoing efforts to strengthen maternal and newborn health services. In Uganda, surveillance data showed that the national incidence of birth asphyxia increased by 4.5% between 2017 and 2020, reaching 31 cases per 1,000 deliveries in 2020 (12). Similarly, in Tanzania, birth asphyxia accounts for approximately 40.6% of neonatal deaths (13). In Kenya, birth asphyxia remains the leading cause of neonatal mortality, contributing to 29% of neonatal deaths (14).

Birth asphyxia is a multifactorial condition resulting from a complex interplay of determinants that compromise fetal oxygenation before, during, or immediately after birth. These determinants can broadly be categorized into maternal, fetal, and obstetric factors. Maternal characteristics associated with birth asphyxia include being a primigravida (15, 16), extremes of age (17, 18), maternal illiteracy (19, 20) and low socioeconomic status (21). Fetal factors include low birth weight (22, 23), prematurity (5, 24), fetal distress (25, 26) and male sex (27, 28). Obstetric factors such as prolonged labour (29, 30), meconium-stained amniotic fluid (3, 24), malpresentations and malpositions (28, 31), inadequate antenatal care (27, 32) and pregnancy-induced hypertension (22, 29) have also been consistently associated with birth asphyxia.

Although Kenya has made policy-level commitments to reduce neonatal mortality to below 12 deaths per 1,000 live births in line with Sustainable Development Goal (SDG) 3.2, the neonatal mortality rate remains high at 20.5 deaths per 1,000 live births (33). To address this burden, the Government of Kenya, through the Ministry of Health has implemented several initiatives to strengthen maternal and newborn services, including the expansion of skilled birth attendance, emergency obstetric and newborn care (EmONC), maternal and perinatal death surveillance and response (MPDSR), quality improvement programmes, and the implementation of national standards for obstetric and newborn care (34–36). Despite these investments, progress in reducing neonatal mortality has been modest, declining from 23 to 21 deaths per 1,000 live births between 2014 and 2024 (37), suggesting that preventable intrapartum-related newborn deaths, including those resulting from birth asphyxia, remain a significant public health challenge. The National Guidelines for Maternal and Perinatal Death Surveillance and Response recognize MPDSR as a quality improvement strategy for determining the causes and modifiable factors associated with maternal and perinatal deaths and implementing corrective actions to prevent future deaths (35). The guidelines further emphasize that strengthening the quality and timeliness of intrapartum and immediate newborn care, identifying health-system failures, and implementing recommended actions are essential for reducing preventable perinatal mortality. However, contemporary evidence on the context-specific determinants of birth asphyxia in high-volume urban public maternity hospitals in Kenya remains limited.

This study therefore aimed to determine the risk factors associated with birth asphyxia in a high-volume urban maternity setting in Kenya. By identifying key maternal, fetal, and obstetric contributors, the findings may provide context-specific evidence to support risk identification, inform quality improvement initiatives and clinical practice, and contribute to national efforts to reduce preventable neonatal morbidity and mortality.

## 2 Methods

### 2.1 Study design

This was a matched case-control study.

### 2.2 Study area

The study was conducted at Pumwani Maternity Hospital, a Level V County Referral Hospital located in Pumwani Ward, Kamukunji Sub-county, Nairobi City County, Kenya. The hospital is situated along General Waruingi Road, adjacent to Starehe Boys’ Centre, approximately 4.5 km from the Nairobi Central Business District, at latitude −1.28028° and longitude 36.84552° (38). Established in 1926, the hospital has evolved into a modern facility providing comprehensive antenatal, labour and delivery, postnatal, neonatal and family planning services. The facility primarily serves low-income populations from surrounding informal settlements, while also receiving referrals from across Nairobi City County. It is a teaching hospital for medical, nursing, and allied health students from various training institutions.

Pumwani Maternity Hospital has 370 inpatient beds, and two dedicated maternity theatres (39). The Newborn Unit (NBU) has 144 baby cots and provides care for both term and preterm neonates requiring specialized neonatal services. The multidisciplinary team comprises nurses, medical officers, obstetricians, gynecologists, neonatologists, pediatricians, pharmacists, and laboratory personnel. Clinical care is guided by the Kenya Ministry of Health Basic Paediatric Protocols and national newborn care guidelines. The hospital records approximately 1,800 to 2,000 deliveries and 380 to 400 newborn admissions to the NBU each month.

### 2.3 Study population

All newborns admitted in Pumwani Maternity Hospital between February to September 2024 constituted the study population. For the purposes of this study, c*ases* were defined as newborns with an APGAR score of < 7 at the fifth minute while c*ontrols* were newborns with an APGAR score of ≥ 7 at the fifth minute. This operational definition was adopted because the five-minute APGAR score was routinely documented for all newborns, whereas umbilical cord arterial blood gas analysis, prolonged resuscitation data, and standardized neurological assessments for hypoxic-ischaemic encephalopathy were not consistently available.

#### 2.3.1 Inclusion criteria

Newborns were eligible for inclusion if they:

- Were admitted to the NBU during the study period.
- Had a gestational age of 28 weeks or more.
- Had a documented five-minute APGAR score.
- Had a mother aged 15 years or older who provided written informed consent.
- Had a mother who was hemodynamically stable, remained admitted to the hospital, and was available for interview at least 24 h after delivery.

#### 2.3.2 Exclusion criteria

Newborns were excluded if they:

- Had major congenital anomalies known to significantly affect neonatal adaptation and survival.
- Had incomplete or missing maternal or neonatal records for variables required in the study.

### 2.4 Participant recruitment

All newborns admitted to the NBU at Pumwani Maternity Hospital during the study period were screened consecutively for eligibility using the predefined inclusion and exclusion criteria. Eligible newborns were classified as cases or controls based on their five-minute APGAR scores. Mothers of eligible newborns were approached for recruitment at least 24 h after delivery to allow adequate time for clinical stabilization and recovery. The purpose of the study, study procedures, potential risks and benefits, and participants’ rights were explained, after which written informed consent was obtained from those who agreed to participate. For mothers who were unable to read or write, the consent form was read aloud in their preferred language, and consent was documented using a thumbprint in the presence of an independent witness.

To minimize emotional distress, recruitment was conducted with support from the hospital counsellor whenever necessary, particularly for mothers of newborns with birth asphyxia. Data were collected in a private room within the NBU at a time convenient for the mother and that did not interfere with routine clinical care. Participation in the study did not influence the clinical management of either the mother or the newborn, and all participants continued to receive standard care according to hospital protocols.

### 2.5 Sample size

The sample size was determined using the matched case-control study formula in Epi-Info^TM^ version 7.2 (Centers for Disease Control and Prevention, Atlanta, GA, USA). The calculation assumed a two-sided 95% confidence level, 80% statistical power, a 1:1 case to control ratio, and a 10% non-response rate. Primiparity was selected as the reference exposure variable because, among the candidate risk factors considered during study planning, it yielded the largest required sample size, thereby ensuring adequate statistical power for evaluating the range of maternal, obstetric, and fetal factors included in the study. The sample size estimation was based on effect estimates reported by Lemma et al. (22), which showed that 36.4% of controls and 48.9% of cases were primiparous, corresponding to an odds ratio of 1.67. Based on these assumptions, the minimum required sample size was 540 participants, comprising 270 cases and 270 matched controls.

### 2.6 Sampling

Consecutive sampling was used to recruit cases into the study, whereby every eligible newborn with an APGAR score of <7 at 5 min was enrolled until the required sample size was achieved. For each case, one eligible control with a five-minute APGAR score of ≥7 was recruited from newborns delivered on the same day. Controls were individually matched to cases primarily by maternal age (±2 years). When no eligible same-day control meeting this criterion was available, neonatal birthweight (±200 g) was used as an alternative matching criterion to maintain the 1:1 matching ratio. Consecutive recruitment from the same facility during the same study period, together with the application of uniform eligibility criteria, helped minimize potential selection bias.

### 2.7 Ethical considerations

Ethical approval was obtained from the Aga Khan University Institutional Scientific Ethics and Review Committee (ISERC) *Ref: 2023/ISERC-77 (v5)*. A research permit was obtained from the National Commission for Science and Technology and Innovation (NACOSTI) *License No: NACOSTI/P/23/31920*. Additional approvals were obtained from Nairobi City County Health Board, the Medical Superintendent of Pumwani Maternity Hospital and the ward in-charges prior to the commencement of the study. Written informed consent was obtained from all participating mothers using consent forms available in both English and Kiswahili. Participation was voluntary, and participants were informed of their right to decline or withdraw from the study at any time without affecting the care they or their newborns received. No financial or material compensation was provided for participation, and participation in the study did not alter the routine clinical care provided to mothers or their newborns. To protect confidentiality, participants’ personal identifiers were removed and replaced with unique study identification numbers. The study was conducted according with the principles of the Declaration of Helsinki.

### 2.8 Data management and analysis

The data collection instruments comprised a researcher-administered questionnaire and a data abstraction tool developed by the study team following a review of the literature on birth asphyxia and its associated risk factors. The instruments were designed to capture maternal sociodemographic characteristics, obstetric history, intrapartum events, fetal characteristics, and neonatal outcomes relevant to the study objectives. Prior to data collection, the instruments were reviewed by the research team to ensure content validity, clarity, completeness, and alignment with the study objectives.

Completed questionnaires and data abstraction forms were entered into separate Microsoft Excel spreadsheets. The datasets were reviewed for completeness, consistency and accuracy, cleaned, and subsequently exported to RStudio, where they were merged using unique participant identifiers, coded, and prepared for statistical analysis.

Descriptive and inferential analyses were conducted. Categorical variables were summarized using frequencies and percentages, while continuous variables were summarized using median and interquartile ranges (IQRs). Descriptive analyses were used to summarize participant characteristics. Bivariate analyses were performed to compare maternal, obstetric, and fetal characteristics between cases and controls. Associations between categorical variables and birth asphyxia were assessed using the Chi-square test or Fisher's exact test, as appropriate, while continuous variables were compared using the Wilcoxon rank-sum test. Univariable conditional logistic regression analysis was then performed to estimate crude odds ratios (ORs) and 95% confidence intervals (CIs). Variables with a *p*-value <0.25 in the univariable conditional logistic regression analysis, together with variables considered clinically or epidemiologically important, were included in the multivariable conditional logistic regression model to account for the matched case-control study design. Model adequacy was assessed using likelihood ratio tests, while predictor variables were evaluated for multicollinearity prior to fitting the final model.

No incomplete records or missing data were identified for the variables included in the final analysis; therefore, no data imputation or other methods for handling missing data were required. Statistical significance was set at *p* < 0.05. All analyses were conducted using R version 4.5.1 (2025-06-13 ucrt).

## 3 Results

### 3.1 Socio-demographic characteristics

Overall, the median maternal age was 24 years (IQR: 21–29), with most participants aged above 20 years (*n* = 433; 80.2%). The majority of mothers were married or cohabiting with a partner (*n* = 408; 75.6%), identified as Christians (*n* = 515; 95.4%), and resided in urban areas (*n* = 504; 93.3%). Regarding maternal education, 105 (19.4%) had attained primary education, 313 (58.0%) secondary education, and 122 (22.6%) tertiary education. Similarly, among fathers, 75 (13.9%) had primary education, 270 (50.0%) secondary education, and 195 (36.1%) tertiary education. More than half of the mothers were unemployed (*n* = 334; 61.9%), and 248 (45.9%) reported a monthly household income of at least KES 4,000 per month. None of the socio-demographic characteristics differed significantly between cases and controls. The socio-demographic characteristics of cases and controls are summarized in Table 1.

**Table 1:** Maternal socio-demographic characteristics of participants in a matched case-control study of birth asphyxia at Pumwani Maternity Hospital, Nairobi, Kenya, February to September 2024.

		Birth asphyxia status		
Characteristic	Overall *N* = 540	Control *N* = 270	Case *N* = 270	*p*-value
Maternal age (years), median (IQR)	24 (21.0–29)	24 (21.0–29)	25 (21.0–30)	0.59
Maternal age category, *n* (%)				0.59
20 years and below	107 (19.8)	56 (20.7)	51 (18.9)	
Above 20 years	433 (80.2)	214 (79.3)	219 (81.1)	
Marital status, *n* (%)				0.84
Single or Divorced	132 (24.4)	67 (24.8)	65 (24.1)	
Married or Living together	408 (75.6)	203 (75.2)	205 (75.9)	
Religion, *n* (%)				0.54
Christian	515 (95.4)	259 (95.9)	256 (94.8)	
Muslim	25 (4.6)	11 (4.1)	14 (5.2)	
Place of residence, *n* (%)				0.49
Urban	504 (93.3)	250 (92.6)	254 (94.1)	
Rural	36 (6.7)	20 (7.4)	16 (5.9)	
Mother's education level, *n* (%)				0.36
Primary education	105 (19.4)	46 (17.0)	59 (21.9)	
Secondary education	313 (58.0)	162 (60.0)	151 (55.9)	
Tertiary education	122 (22.6)	62 (23.0)	60 (22.2)	
Father's education level, *n* (%)				0.79
Primary education	75 (13.9)	35 (13.0)	40 (14.8)	
Secondary education	270 (50.0)	138 (51.1)	132 (48.9)	
Tertiary education	195 (36.1)	97 (35.9)	98 (36.3)	
Mother's occupation, *n* (%)				0.38
Unemployed	334 (61.9)	162 (60.0)	172 (63.7)	
Employed	206 (38.1)	108 (40.0)	98 (36.3)	
Monthly household income, *n* (%)				0.82
No Income	249 (46.1)	123 (45.6)	126 (46.7)	
KES 1 - 3900(<$1 per day)	43 (8.0)	20 (7.4)	23 (8.5)	
Above KES. 3900 (>$1 per day)	248 (45.9)	127 (47.0)	121 (44.8)	

IQR, Interquartile Range, KES, Kenya Shillings.

### 3.2 Maternal, obstetric and fetal factors

There were significant differences between cases and controls in several maternal, obstetric and fetal characteristics as summarized in Table 2. Cases were more likely than controls to experience complications during labour (96.7% vs. 69.3%, *p* < 0.001), and maternal medical or obstetric conditions during pregnancy (74.8% vs. 60.7%, *p* < 0.001). Spontaneous vaginal delivery was less frequent among cases than controls (80.4% vs. 97.4%), whereas caesarean section delivery was more common among cases (19.6% vs. 2.6%, *p* < 0.001). Significant differences were also observed in fetal presentation at delivery (*p* = 0.002), onset of labour (*p* = 0.009), type of birth attendant (*p* = 0.007), and presence of a nuchal cord (*p* = 0.022). Specifically, unattended deliveries (4.4% vs. 0.7%) and nuchal cord (10.4% vs. 4.8%) were more common among cases than controls. In contrast, time of delivery, number of babies delivered, ANC attendance, and number of ANC visits were not significantly different between cases and controls (*p* > 0.05).

**Table 2:** Maternal, obstetric, and fetal characteristics of participants in a matched case-control study of birth asphyxia at Pumwani Maternity Hospital, Nairobi, Kenya, February to September 2024.

		Birth Asphyxia Status		
Characteristic	Overall *N* = 540	Control *N* = 270	Case *N* = 270	*p*-value
Previous obstetric history, *n* (%)	162 (30.0)	71 (26.3)	91 (33.7)	0.060
Maternal medical/obstetric conditions, *n* (%)	366 (67.8)	164 (60.7)	202 (74.8)	<0.001
Complications during labour, *n* (%)	448 (83.0)	187 (69.3)	261 (96.7)	<0.001
Abnormal vaginal discharge, *n* (%)	151 (28.0)	70 (25.9)	81 (30.0)	0.29
Onset of labour, *n* (%)				0.009
Induced	30 (5.6)	8 (3.0)	22 (8.1)	
Spontaneous	510 (94.4)	262 (97.0)	248 (91.9)	
Nuchal cord, *n* (%)				0.022
Yes	41 (7.6)	13 (4.8)	28 (10.4)	
No	499 (92.4)	257 (95.2)	242 (89.6)	
Mode of delivery, *n* (%)				<0.001
Spontaneous vaginal delivery	480 (88.9)	263 (97.4)	217 (80.4)	
CS	60 (11.1)	7 (2.6)	53 (19.6)	
Birth attendant, *n* (%)				0.007
Student	194 (35.9)	107 (39.6)	87 (32.2)	
Licensed healthcare provider (nurse/midwife, medical officer, or obstetrician)	332 (61.5)	161 (59.6)	171 (63.3)	
Unattended	14 (2.6)	2 (0.7)	12 (4.4)	
Time of delivery, *n* (%)				0.49
Day	288 (53.3)	140 (51.9)	148 (54.8)	
Night	252 (46.7)	130 (48.1)	122 (45.2)	
Fetal presentation at delivery, *n* (%)				0.002
Cephalic	515 (95.4)	265 (98.1)	250 (92.6)	
Non-cephalic	25 (4.6)	5 (1.9)	20 (7.4)	
Number of babies delivered *n* (%)				0.24
Singleton	528 (97.8)	266 (98.5)	262 (97.0)	
Multiple gestation	12 (2.2)	4 (1.5)	8 (3.0)	
Attended ANC (yes/no), *n* (%)	526 (97.4)	262 (97.0)	264 (97.8)	0.59
Number of ANC visits, *n* (%)				0.33
<4 ANC Visits	146 (27.0)	78 (28.9)	68 (25.2)	
4 or more ANC Visits	394 (73.0)	192 (71.1)	202 (74.8)	

ANC, Antenatal Clinic.

### 3.3 Risk factors associated with birth asphyxia

In the multivariable analysis, mothers with a previous adverse obstetric history had more than twice the odds of delivering a baby with birth asphyxia than those without such history (AOR: 2.18; 95% CI: 1.14–4.17). Similarly, mothers with medical or obstetric conditions during pregnancy had 82% higher odds of birth asphyxia compared to those without such conditions (AOR: 1.82; 95% CI: 1.04–3.19). Complications during labour were strongly associated with birth asphyxia, with affected mothers having more than 24-fold higher odds of delivering a neonate with birth asphyxia than those without labour complications (AOR: 24.26; 95% CI: 7.65–76.95). In addition, non-cephalic fetal presentation was associated with significantly higher odds of birth asphyxia (AOR: 8.92; 95% CI: 1.77–45.00).

Conversely, mothers with spontaneous onset of labour had 80% lower odds of birth asphyxia than those with induced labour (AOR: 0.20; 95% CI: 0.05–0.76). Similarly, spontaneous vaginal delivery was associated with a 98% reduction in the odds of birth asphyxia compared to caesarean section delivery (AOR: 0.02; 95% CI: 0.00–0.10). Delivery by a trained healthcare provider was associated with 73% lower odds of birth asphyxia compared to unattended delivery (AOR: 0.27; 95% CI: 0.14–0.52). Table 3 summarizes the univariable and multivariable conditional logistic regression analyses of factors associated with birth asphyxia.

**Table 3:** Univariable (crude) and multivariable (adjusted) conditional logistic regression analysis of factors associated with birth asphyxia in a matched case-control study at Pumwani Maternity Hospital, Nairobi, Kenya, February to September 2024.

Variable	Level	Crude OR (95% CI)	*p*-value	Adjusted OR (95% CI)	Adj *p*-value
Maternal age (years)		1.01 (0.97–1.06)	0.542	1.02 (0.94–1.12)	0.601
Mother age (continuous)	20 years and below	1.00 (Reference)		—	—
	Above 20 years	1.23 (0.71–2.13)	0.459	—	—
Marital status	Married or Living together	1.02 (0.68–1.54)	0.917	—	—
	Single or Divorced	1.00 (Reference)		—	—
Religion	Christian	1.00 (Reference)		—	—
	Muslim	1.18 (0.53–2.64)	0.683	—	—
Place of residence	Rural	0.80 (0.41–1.58)	0.530	—	—
	Urban	1.00 (Reference)		—	—
Mother's education	Primary education	1.00 (Reference)			
	Secondary education	0.67 (0.41–1.09)	0.110	0.66 (0.27–1.61)	0.363
	Tertiary education	0.73 (0.42–1.24)	0.242	0.48 (0.18–1.27)	0.139
Father's education	Tertiary education	0.89 (0.51–1.53)	0.662	—	—
	primary education	1.00 (Reference)		—	—
	secondary education	0.85 (0.51–1.39)	0.511	—	—
Mother's occupation	Employed	0.84 (0.59–1.21)	0.344	—	—
	Unemployed	1.00 (Reference)		—	—
Mother monthly income	Above KES 3900 (>$1 per day)	0.93 (0.65–1.33)	0.686	—	—
	KES 1 - 3900(<$1 per day)	1.13 (0.58–2.22)	0.715	—	—
	No Income	1.00 (Reference)		—	—
Gestational age (weeks)	Preterm birth	1.00 (Reference)			
	Term birth	1.15 (0.65–2.01)	0.634	0.53 (0.19–1.47)	0.224
Parity	Grand multiparous	0.41 (0.15–1.13)	0.084	0.21 (0.03–1.24)	0.084
	Multiparous	0.79 (0.52–1.18)	0.250	0.47 (0.21–1.06)	0.07
	Primiparous	1.00 (Reference)			
Birth weight	Low birthweight	2.80 (1.17–6.67)	0.020	4.62 (0.88–24.26)	0.071
	Normal birthweight	1.00 (Reference)			
Sex of newborn	Female	0.76 (0.53–1.09)	0.140	0.91 (0.52–1.61)	0.753
	Male	1.00 (Reference)			
Previous obstetric history	No	1.00 (Reference)			
	Yes	1.47 (1.00–2.16)	0.048	2.18 (1.14–4.17)	0.019
Medical/obstetric condition	No	1.00 (Reference)		—	—
	Yes	1.84 (1.28–2.64)	0.001	1.82 (1.04–3.19)	0.037
Complications during labour	No	1.00 (Reference)			
	Yes	13.38 (5.83–30.69)	<0.001	24.26 (7.65–76.95)	<0.001
Abnormal vaginal discharge	No	1.00 (Reference)		—	—
	Yes	1.18 (0.82–1.70)	0.369	—	—
Onset of labour	Induced	1.00 (Reference)		—	—
	Spontaneous	0.29 (0.12–0.71)	0.007	0.20 (0.05–0.76)	0.018
Nuchal cord	No	1.00 (Reference)			
	Yes	2.40 (1.15–5.02)	0.02	1.42 (0.48–4.18)	0.52
Mode of delivery	CS	1.00 (Reference)		—	—
	Spontaneous vaginal delivery	0.04 (0.01–0.18)	<0.001	0.02 (0.00–0.10)	<0.001
Birth attendant	Student or licensed healthcare provider	0.76 (0.52–1.10)	0.141	0.27 (0.14–0.52)	<0.001
	Unattended	5.65 (1.25–25.59)	0.025	6.59 (0.87–50.04)	0.068
Time of delivery	Day	1.00 (Reference)		—	—
	Night	0.89 (0.63–1.25)	0.500	—	—
Fetal presentation at delivery	Cephalic	1.00 (Reference)			
	Non-cephalic	4.75 (1.62–13.96)	0.005	8.92 (1.77–45.00)	0.008
Number of babies delivered	Multiple babies	2.33 (0.60–9.02)	0.220	4.24 (0.42–43.04)	0.222
	Singleton	1.00 (Reference)		1.00 (Reference)	—
ANC attendance	No	1.00 (Reference)		—	—
	Yes	1.33 (0.46–3.84)	0.594	—	—
Number of ANC visits	4 or more ANC Visits	1.21 (0.82–1.77)	0.332	1.33 (0.71–2.50)	0.371
	<4 ANC Visits	1.00 (Reference)			

ANC, antenatal clinic, CI, confidence interval, CS, caesarian section, KES, Kenya shillings, OR, odds ratio.

— Variable not included in the final multivariable conditional logistic regression model.

## 4 Discussion

This matched case-control study identified risk factors independently associated with birth asphyxia in a high-volume urban maternity setting in Kenya. Complications during labour, previous adverse obstetric history, maternal medical and obstetric conditions during pregnancy, and non-cephalic fetal presentation were associated with increased odds of birth asphyxia. Conversely, spontaneous onset of labour, spontaneous vaginal delivery, and delivery conducted by trained healthcare providers were associated with lower odds of birth asphyxia.

Complications during labour were the strongest factor associated with birth asphyxia in this study, with affected mothers having more than 24-fold higher odds of delivering a neonate with birth asphyxia. This finding is consistent with studies from Ethiopia (29), Uganda (28), and Colombia (40), which similarly reported higher odds of birth asphyxia among women who experienced labour-related complications such as meconium-stained liquor, prolonged labour, obstructed labour, instrumental delivery, and non-reassuring fetal status. Labour complications compromise uteroplacental blood flow and fetal oxygenation, thereby increasing the likelihood of intrapartum hypoxic injury (41, 42). The particularly strong association observed in this study may reflect the severity of these obstetric complications, which require prompt recognition and timely intervention to prevent fetal hypoxia. In high-volume public maternity facilities such as Pumwani Maternity Hospital, delays in diagnosis or definitive management, coupled with overcrowding and resource constraints, may further amplify this risk. These findings highlight the importance of continuous labour monitoring using the partograph and timely escalation of obstetric care when complications arise.

Maternal medical and obstetric conditions during pregnancy were also significantly associated with birth asphyxia, with affected mothers having nearly twice the odds of delivering a baby with birth asphyxia compared with mothers without such conditions. This finding is similar to those reported in studies from Ethiopia and Uganda, where maternal conditions including hypertensive disorders of pregnancy, antepartum haemorrhage, anaemia, maternal infections, and gestational diabetes were associated with higher odds of birth asphyxia (2, 28, 43, 44). These maternal conditions may impair uteroplacental perfusion, contribute to fetal growth restriction, precipitate preterm labour, and necessitate emergency obstetric interventions, all of which increase the risk of intrapartum fetal hypoxia (45). In settings where maternal complications are not identified or managed promptly, these adverse physiological effects may further increase the likelihood of birth asphyxia.

Women with a previous adverse obstetric history had more than twice the odds of delivering a baby with birth asphyxia compared with those without such a history. The association between previous adverse obstetric history and birth asphyxia observed in this study may reflect the cumulative influence of underlying maternal and reproductive health vulnerabilities. Women with adverse obstetric histories are more likely to experience recurrent pregnancy and labour complications that predispose neonates to intrapartum distress (46, 47). Similar findings have been reported in studies from South India (48) and Tanzania (49), where previous obstetric complications increased the likelihood of birth asphyxia. This highlights the importance of comprehensive obstetric risk assessment during antenatal care, particularly for women with a history of adverse pregnancy outcomes, to facilitate closer monitoring and timely interventions.

Non-cephalic fetal presentation was another important predictor of birth asphyxia in this study. This finding is consistent with reports from Ayebare et al. (28), Mergia and Melaku (50) and Worke et al. (51) where breech and other malpresentations were similarly associated with an increased risk of birth asphyxia. Non-cephalic presentations often complicate the normal mechanisms of labour, increasing the risk of prolonged or obstructed labour, umbilical cord compression, and fetal distress, all of which may compromise fetal oxygenation and increase the likelihood of intrapartum hypoxic injury (24, 31). Inadequate intrapartum monitoring coupled with delayed obstetric intervention may further worsen neonatal outcomes in pregnancies complicated by fetal malpresentation. These results emphasize the importance of early identification of fetal presentation during antenatal care and vigilant intrapartum monitoring to facilitate timely obstetric intervention.

Spontaneous onset of labour and spontaneous vaginal delivery were associated with lower odds of birth asphyxia in this study. Evidence comparing spontaneous and induced labour in relation to neonatal outcomes remains inconsistent. While elective induction has been associated with improved neonatal outcomes in some settings (52–54), other studies have demonstrated more favourable maternal and neonatal outcomes among women with spontaneous onset of labour compared with those undergoing induction (55, 56). The association observed in this study may reflect the fact that spontaneous labour is generally a physiological process associated with lower rates of labour complications and emergency obstetric interventions (57). Similarly, spontaneous vaginal delivery is often achieved in pregnancies without major maternal or fetal complications, whereas operative deliveries are frequently undertaken because of intrapartum complications or fetal compromise, both of which increase the risk of birth asphyxia. In addition, vaginal birth promotes physiological adaptation to extrauterine life, including clearance of fetal lung fluid during passage through the birth canal, which facilitates effective neonatal respiratory transition after birth (23, 41).

Although caesarean section is often a lifesaving intervention in obstetric emergencies, the higher odds of birth asphyxia among neonates delivered by caesarean in this study likely reflect the underlying fetal compromise and labour complications necessitating surgical delivery rather than the procedure itself. Previous studies have similarly reported an increased risk of birth asphyxia among neonates delivered by emergency caesarean section, attributing this association to factors such as prolonged decision-to-delivery intervals, fetal distress, prolonged labour, and delayed obstetric intervention (58, 59). However, this association is not consistently observed, as Udu et al. (60) found no significant association between the timing of caesarean section and birth asphyxia. Taken together, these findings suggest that neonatal outcomes may be influenced more by the severity of the underlying obstetric condition and the timeliness of obstetric care than by the mode of delivery itself.

Delivery conducted by trained healthcare providers was associated with lower odds of birth asphyxia in this study compared to unattended deliveries. This finding is consistent with previous studies from sub-Saharan Africa, which have shown that inadequate intrapartum monitoring and lack of skilled birth attendance contribute substantially to preventable birth asphyxia and neonatal mortality (14, 61). Skilled birth attendants are better equipped to recognize deviations from normal labour, initiate timely obstetric interventions, and provide effective neonatal resuscitation when required (62). The association observed in this study likely reflects the capacity of skilled providers to detect and manage intrapartum complications before they progress to severe fetal compromise. In high-volume maternity settings such as Pumwani Maternity Hospital, strengthening staffing capacity, continuous mentorship, and emergency obstetric and newborn care preparedness may further improve early recognition and management of labour complications, thereby reducing the burden of birth asphyxia.

## 5 Strengths and limitations

The primary strength of this study is its matched case-control design, which minimized confounding between cases and controls and enabled the assessment of multiple maternal, obstetric, and fetal factors associated with birth asphyxia. The large sample size of 540 neonates and high participation rate strengthened the statistical power and precision of our estimates. In addition, the use of both structured interviews and medical record reviews enhanced the reliability of the findings by allowing triangulation of maternal self-reported information with clinical data. Standardized data collection tools, together with trained research personnel, further minimized information bias. Furthermore, conditional multivariable logistic regression was used to adjust for potential confounding variables, thereby strengthening the validity of the associations identified.

Despite these strengths, the study had some limitations. First, birth asphyxia was operationally defined using a five-minute APGAR score, which, although widely used in epidemiological studies and routine clinical practice, does not by itself establish intrapartum hypoxia. A comprehensive diagnosis of birth asphyxia also considers additional parameters such as umbilical cord arterial blood gas analysis, the need for prolonged resuscitation, evidence of hypoxic-ischaemic encephalopathy, and multisystem organ dysfunction, none of which were consistently available in the study setting. Second, although consecutive recruitment and uniform eligibility criteria were used to minimize selection bias, the study included only newborns admitted to the NBU and mothers who were available to participate after delivery. Consequently, newborns who died before admission, were transferred elsewhere, or whose mothers were too ill to participate may have been underrepresented. Third, the study was conducted in a single referral facility, which may limit the generalizability of findings to rural settings and lower-level health facilities. Nevertheless, the study provides important context-specific evidence on modifiable maternal and obstetric factors associated with birth asphyxia in a major public referral maternity hospital in Kenya.

## 6 Conclusions

Birth asphyxia in this study was strongly associated with several maternal, and obstetric factors, particularly complications during labour, maternal medical and obstetric conditions, previous obstetric history, and non-cephalic fetal presentation. Conversely, spontaneous onset of labour, spontaneous vaginal delivery, and delivery conducted by trained healthcare providers were associated with lower odds of birth asphyxia. These findings highlight the importance of strengthening antenatal risk identification, skilled intrapartum care, timely obstetric intervention, and effective labour monitoring to reduce the burden of birth asphyxia and improve neonatal outcomes.

## Data Availability

The raw data supporting the conclusions of this article will be made available by the authors, without undue reservation.
